# Evidence-based yet still challenging! Research on physical activity in old age

**DOI:** 10.1186/s11556-023-00318-3

**Published:** 2023-03-17

**Authors:** Michael Brach, Eling D. de Bruin, Oron Levin, Timo Hinrichs, Wiebren Zijlstra, Yael Netz

**Affiliations:** 1 Institute of Sport and Exercise Sciences, University of Münster, Münster, Germany; 2 Department of Health, OST – Eastern Swiss University of Applied Sciences, St. Gallen, Switzerland; 3 Institute of Human Movement Sciences and Sport, Department of Health Sciences and Technology, ETH Zurich, Zurich, Switzerland; 4 Department of Neurobiology, Care Sciences and Society, Karolinska Institute, Stockholm, Sweden; 5 Movement Control and Neuroplasticity Research Group, Biomedical Sciences, KU Leuven, Louvain, Belgium; 6 Division of Sports and Exercise Medicine, Department of Sport, Exercise and Health, University of Basel, Basel, Switzerland; 7 Institute of Movement and Sport Gerontology, German Sport University Cologne, Cologne, Germany; 8 Levinsky-Wingate Academic Center, The Academic College at Wingate, Netanya, Israel; 9 Department of Health Promotion and Rehabilitation, Lithuanian Sports University, Kaunas, Lithuania

## Abstract

Preserving functional health and quality-of-life in old age is a major goal and global challenge in public health. The high rate of sedentary behavior that is characteristic of the older adult population exacerbates impairments of physiological and structural systems that are typically seen in the aging process. Achieving an understanding of the profound influence of physical activity on all aspects of health in old age is the driving force behind the emergence of "physical activity in old age" as a growing area of research. Accumulated evidence implies that being physically active and exercising is far superior to other optimal aging facilitators. Yet this area of research faces numerous constraints and obstacles. This commentary addresses some of these challenges, primarily the heterogeneity of the aging process, which induces both inter- and intra-individual differences among aged individuals, heterogeneity in assessment tools, unjustified inclusion/exclusion criteria and insufficient recruitment strategies, difficulties in implementing research results in real-world conditions, and rudimentary exploitation of innovative technology. We explain the importance of establishing a network of multidisciplinary scientists and stakeholders to propose consensus-based goals and scientifically evidenced wide-ranging plans for dealing with these challenges. In addition, we suggest work directions for this network.

## Introduction

The vast corpus of research on physical activity (PA) in advanced age covers numerous domains, yet consistently indicates the health benefits of performing PA (e.g., see reviews on cognition [1]; depression [2]; 26 chronic diseases [3]; mortality [4, 5]; and the immune system [6]. See also The Copenhagen Consensus Statement 2019: PA and ageing [7]).

The literature usually distinguishes between PA and exercise, whereby the term PA refers to any bodily movement that is produced by the skeletal muscles and that increases energy expenditure compared to resting [7]. This may include structured and unstructured forms of leisure, transport, and domestic or work-related activities. The intensity and duration of PA may also vary substantially. The term *exercise* is a subcategory of PA, one that is planned, structured, and repetitive, and is more specifically designed to improve certain fitness components, such as cardiorespiratory fitness, flexibility, balance, coordination, strength, and/or power [7, 8].

The "Exercise is medicine" slogan, stated by the American College of Sports Medicine in 2007 (https://www.exerciseismedicine.org), has become a global health initiative, especially in relation to healthy aging (e.g., [9]). Subsequently, PA has long since been included in mainstream health behavior recommendations for older ages [8, 10]. Yet scientists still face challenges in translating research into practice and policy. Indeed, the task of integrating the existing body of knowledge into solid conclusions is often perplexing, as is the transferring of such knowledge from the laboratory environment and other artificial settings to real-world ones. For example, The World Health Organization’s (WHO) official guidelines on PA and sedentary behavior call for at least 150–300 min of moderate-to-vigorous-intensity PA per week [10]. However, this “one size fits all” regime may not encompass specific issues, such as responsiveness to PA [11], as not all individuals respond to PA in a positive manner. As such, health professionals may have to create more individually-focused PA programs that are designed to overcome non-responsiveness [11]. Another example is the widely promoted “10,000 steps per day,” which has recently been challenged by scientific evidence. Interestingly, one of the findings is that for older women, the mortality risk plateau is only 7500 steps per day [12]. Such gaps in the generalization of findings may stem from heterogeneity in protocols and assessment tools [13].

The main aim of this commentary is to describe some of these challenges, while highlighting their complexity. Subsequently, we propose combining the efforts of multidisciplinary scientists and practitioners, including but not limited to the fields of exercise sciences, geriatrics and gerontology, life sciences, behavioral sciences, health disciplines, and computer sciences. Such a multi-disciplinary network will be able to generate and publish literature-supported guidelines, standards, and frameworks in the field of PA for older adults. A secondary aim of this commentary is to offer recommendations for this network of professionals to begin the process.

## Challenges

### Heterogeneity in the aging process

Compared to other age groups, old age is typified by increased heterogeneity, which complicates research [14] and renders practice harder to conduct [15]. This heterogeneity is typical of physical performance (e.g., [16]), as well as cognitive (e.g., [17]) and behavioral (e.g., [18]) performance.

Evidence shows that aging is associated with increases in both inter- and intra-individual differences [17, 19, 20]. Inter-individual differences in old age stem from a range of causes, including genetics, age-related diseases, cohort effects, and most importantly – differences in lifestyles, including differences in habitual PA [21]. Yet even more complex for research and practice are the intra-individual variations [20], which are associated with the measuring of a single person in either multiple tasks (dispersion) or in a single task yet on multiple occasions (inconsistency) [17]. Such inter- and intra-individual differences have also been reported in response to PA, yet scientific understanding is still limited with regards to the characteristics that could help identify responders and non-responders [15, 22, 23]. Identifying principal biological and behavioral markers of responsiveness to PA interventions could significantly enhance clinicians' ability to prescribe PA in a more individualized and effective manner [15].

Notably, external environmental factors, such as stairs, large distances to services, inadequate walkways, or insufficient means of transportation, are meaningful moderators in habitual PA in older age [24, 25]. As such, they should be incorporated in the determinants of PA behaviors and related recommendations. In addition to the heterogeneity that is inherent in the complex aging process, and typical of all cultures and ethnic groups, socio-cultural differences can be seen between and within geographical regions. The cultural environment may also affect PA behavior, and as such, should be addressed when making PA recommendations [26, 27].

A more recent approach to heterogeneity in aging relates to the functional complexity of the individual’s various physiological and organ systems. It has been argued that basic physiology widely employs a reductionist approach, addressing human functioning through the prism of individual organ systems. Yet the human organism is a complex and integrated network, where multi-component physiological systems continuously interact, to coordinate their functions [28].

With respect to PA in old age, a reductionist approach is often adopted. For example, PA programs may be developed to affect specific types of performance (e.g., muscle strength, aerobic endurance, and balance), by manipulating parts of an intact physiological movement system. However, one typical manifestation of aging is a heterogenous decline in these physiological systems. In healthy organs, these systems communicate with one another to maintain homeostasis; yet aging causes the breakdown of different physiological systems, which in turn may affect other more intact physiological systems, thereby interfering with this homeostasis. As such, this individual pattern of aging could imply the need for critically rethinking PA training principles for aging populations. As such, instead of focusing on improving performance through a reductionist approach, implementing a system approach may be more suitable when designing PA programs for older adults [21, 29].

### Heterogeneity in assessment tools and interventions

Due to the large heterogeneity of evaluation methods and PA interventions, standardized guidelines are lacking for assessing the benefits of PA programs for specific groups of older adults [13]. While the literature presents numerous reports that employed specific criteria for assessing physical fitness, the PA programs that were used for such interventions are not always clearly described. Moreover, in addition to the lack of standardized assessment tools and intervention details, research studies fall short in providing guidelines for the standardized reporting of PA protocols – in terms of the type of PA, intensity, number of repetitions, and more. The related difficulties are twofold. First, it is difficult for other researchers to reproduce findings or evaluate a successful protocol in different populations or settings. Moreover, it is difficult for exercise professionals and other stakeholders to apply or disseminate a successful protocol in a target group.

Scientific communities have embraced initiatives relating to evidence-based medicine (EBM) as a means for developing guidelines that could help improve the design, conduct, and reporting of interventional research. These include checklists and statements on how to develop a research protocol and how to report study designs and results. EBM has substantially contributed to improving research quality, by transparently documenting issues regarding existing research and subsequently developing enhanced research standards. EBM has also improved the practice of medicine, by developing methods and techniques for generating systematic reviews and clinical practice guidelines [30]. In exercise sciences, however, there is a dearth of evidence-based strategies that are applied in the designing, conducting, and reporting of intervention studies. Researchers encounter various conceptual challenges and pitfalls that are related to research designs, interventional approach characteristics, control conditions and groups, reliable and valid measures, sample sizes, statistical approaches to data analysis, and more [31]. Various types and forms of PA, including aerobics, resistance or balance training, dance, yoga, Pilates, and flexibility, are examples of interventions that have been found to be effective in the prevention and treatment of a range of acute and chronic conditions, yet that have been poorly described in randomized clinical trial reports in aging populations [32]. It therefore seems justified to assume that consistent reporting guidelines [32–34] could have led to notable improvements in this field.

### Unjustified exclusion criteria and insufficient recruitment strategies

With exercise and other types of PA, there is clearly no “one-size-fits-all,” especially in the diverse aged population. Yet, research on older populations is typically biased towards healthy and relatively young older adults [35], with certain groups of older individuals frequently being excluded from research on aging – especially in studies with PA interventions [36]. This seems to stem from a range of factors, including inadequate recruitment strategies [36, 37]; individuals may even be excluded based on unjustifiable and therefore unethical upper age limits [38]. More specifically, barriers may include communication issues and cognitive difficulties, limited mobility, transportation difficulties, low income, and even self-imposed ageism [35–37]. Such practices hinder the generalization of research findings, as well as the development of evidenced-based PA interventions. The inclusion of individuals with age-related pathologies and/or disabilities is expected to enlarge existing databases, creating more solid ground for applying the EBM approach in relation to PA programs for older adults [39]. Furthermore, such inclusion would also fulfill an acknowledged need that stems from demographic shifts, specifically the anticipated disproportionate growth of this population segment in the near future [40–43].

### Transitioning from highly controlled studies to real-world environments

Despite evidence on the benefits of PA in advanced age, public health initiatives often fail to examine clinically relevant effects of PA on physical and cognitive health (e.g., [44]). For example, it has been hypothesized that the highly controlled environments in which some PA research is conducted limit its replicability in real-world community settings [45]. While the efficacy of the PA intervention may perhaps be more clearly demonstrated in laboratory settings, there is a dearth of research that indicates its effectiveness when conducted in real-world conditions as well [46]. In addition, the effect of PA in clinical populations may vary, based on the stage of the disease, the nature of a concomitant medical treatment, and the patients’ current lifestyle [47].

Moreover, studies often stop at the evaluation stage, resulting in scarce research at the implementation stage – as recently pointed out in relation to cardiovascular rehabilitation [48]. Complementary to this issue is the complex interventions topic that was recently updated by the UK Medical Research Council [49]: "Complex intervention research goes beyond asking whether an intervention works in the sense of achieving its intended outcome—to asking a broader range of questions (e.g., identifying what other impact it has, assessing its value relative to the resources required to deliver it, theorizing how it works, taking account of how it interacts with the context in which it is implemented, how it contributes to system change, and how the evidence can be used to support real world decision making)" [49 p.1]. Research on PA in old age, as discussed in this commentary (e.g., population diversity, methodological limitations, and heterogeneity in assessment tools and interventions) certainly falls under the category of complex interventions.

### Rudimentary exploitation of innovative technology, big data, and open data in PA research and practice

#### Innovative technology

The past few decades have seen a growing interest in the application of technological innovations for assessing, promoting, and assisting with PA interventions. This includes the use of novel devices, software, and wearable technologies, such as activity trackers (e.g., [50]), body-worn sensors (e.g., [51]), mobile phone applications (e.g., [52]), tablets (e.g., [53]), virtual reality [54], exergames (e.g., [55]), global positioning system (GPS) devices, and map-based tools (e.g., [56]). Gamification of exercise programs, for example, enables the targeting of both physical functions and cognitive ones, and allows users to perform their training programs in a location of their convenience, while being remotely monitored and/or supervised [57]. Although the large potential of such approaches has been acknowledged, there is a need for the filling of research gaps [58].

A recent systematic review on adherence to technology-based exercise programs in older adults indicates that technology offers a well-accepted method for providing older adults with exercise opportunities, and that adherence to such programs is high [59]. Yet small sample sizes, short follow-up periods, inclusion of mostly healthy older people, and problematic methods-related issues that are used for reporting PA adherence – all limit the generalizability of these findings [59]. As such, a critical methodological issue relates to the validation of new devices. Indeed, the creation of widely accepted validation protocols has been suggested, to enable comparisons between devices and in relation to defined activities, cohorts, and environments. Using the optimal system for a described cohort of older persons, relevant PA databases could be established [60].

#### PA databases (big data) and artificial intelligence

Understanding the underpinning mechanisms of PA behavior and responses to exercise at the cellular and molecular level is a necessary yet insufficient step, as it must then be translated into individually tailored programs. The emergence of databases that contain electronic health records, biobanks, and more focused long-term cohort studies has created greater opportunities for understanding these mechanisms [22]. Innovative technologies such as longitudinal multi-omics profiling, combined with clinical, functional, and behavioral measures, could comprehensively assess health biomarkers. Yet few studies have leveraged emerging technologies and longitudinal profiling for managing health markers [61]. While quite a few studies have been published based on big data sources (e.g., the UK biobank study [62] and the China Kadoorie Biobank study [63]), they tend to investigate PA in relation to specific conditions and diseases [64]. Moreover, in these studies, PA serves as one of many independent variables and moderators for predicting health, yet not as an outcome that is derived from all other variables for determining optimal guidelines for PA in the diverse population of older adults.

Recent research conducted by the FARSEEING consortium (e.g., see [65]) addresses falls in older people, by developing a data repository that is founded on novel sensor-based technologies that monitor PA and falls in real-life. Through collaborative efforts made by multiple international research groups, the work has resulted in a large-scale database that allows data sharing and analysis of real-life falls and PA. Ultimately, the research aims at improving the understanding of real-life falls, while enabling new approaches to fall prevention in older people. It could be expected that similar collaborative efforts, based on novel technologies, standardized PA data collection, and data sharing could provide the foundation for a better understanding of health-related benefits of PA.

The recently established prestigious National Institute of Health (NIH) project, MoTrPAC, aims at mapping the molecular transducers that are involved in responses to exercise. The project declares that: “Exercise provides a robust physiological stimulus that evokes cross-talk among multiple tissues that when repeated regularly improves physiological capacity, benefits numerous organ systems, and decreases the risk for premature mortality. However, a gap remains in identifying the detailed molecular signals induced by exercise that benefit health and prevent disease” ([66] p 1484). In turn, our goal is to compile these big data sources and artificial intelligence (AI) models, to create optimal PA programs for diverse older adult populations.

#### Open data

Further progress will be facilitated by collaboration between scientists in these different fields. Doing so will align efforts to test the effect of feasible PA interventions on aging biomarkers, hallmarks, multimorbidity, and frailty at the individual level [22].

## Recommendations

In relation to the challenges presented above, certain ideas, options, and even elaborated recommendations for improving research already exist (e.g., [67, 68]). While some can be implemented in their current format, others require certain adaptions to the particular field of PA or to the specific population of older adults. Others still may require new concepts and development.

Based on thorough reviews of the literature, solutions require input from various disciplines, from clinical and real-world knowledge, and from different user groups. Clearly, the complexity of the challenges requires in-depth and ongoing groundwork that is conducted by a network of scientists. Consequently, our main recommendation is to establish a network that strives to overcome the challenges that are presented in this commentary, while considering the following recommendations:

(a) To identify and classify biomarkers and behavioral markers regarding cognitive and motor performance, fitness, sedentary and mobility behavior, and frailty in the aging population. For example, building a platform that includes data regarding all known markers that are involved in PA and movement in advanced age. Doing so will enable the generating of a molecular, cellular, or cerebral map for exploring the underlying mechanisms that mediate between various organ systems and PA in advanced age. This will also enable the mapping of cross-talks between networks across all PA modes, levels of functioning, and health conditions. An additional example can be seen in the creating of a platform of data that are derived from living (on-going) systematic reviews and that explore the effect of various modes of PA on brain and blood markers, as well as on environmental, emotional, and social ones, in diverse groups of older adults. Doing so will significantly enhance clinicians’ ability to prescribe PA in a more individualized and effective manner.
  (b) To initiate novel approaches to aging heterogeneity and functional complexity. For example, since the human organism is an integrated network in which multi-component physiological systems constantly interact to coordinate their functions, a network approach should be taken when researching physical exercise, rather than the reductionist approach of examining individual body organs. This could, for example, mean that training programs that target physical frailty should not reduce their focus to a single physiological system (e.g., aerobic exercises for improving cardiovascular functioning) [69]. Instead, interventions should focus on improved communications within and between the metabolic, stress-response, and musculoskeletal systems – three physiological systems that are believed to be responsible for declined physical functioning with age [70].
  To establish guidelines for study reports, intervention protocols, measurement standardization, and evaluation tools among research groups. Guidelines exist for reporting findings of studies in the clinical health studies (e.g., CONSORT Checklist for randomized controlled studies), or guidelines for defining the boundaries of a systematic review (PICO – Participants, Interventions, Comparators, Outcomes). There are also general platforms such as Equator (https://www.equator-network.org/toolkits/developing-a-reporting-guideline) or Fair sharing (FAIRsharing.org) which gather all reporting guidelines in one website. These guidelines are recommended for authors in most health journals, including those on PA in old age (e.g., the European Review of Aging and Physical Activity – EURAPA, and Journal of Aging and Physical Activity – JAPA). It is possible that for studies on PA and on exercise, different reporting checklists are needed, including specific guidelines for describing the interventions, the functional status of the participants, and more. A recent novel approach to conducting systematic reviews in health sciences is the meta-analytical research domain [71], whereby there is a need to progress from meta-analysis to higher levels of aggregation of RCTs (randomized controlled trails) outcomes. In other words, by expanding reviews to include numerous PICOs, broader conclusions can be drawn – to encompass the domain in question in a more comprehensive manner. Conducting such meta-analysis seems a reasonable means for providing answers to basic questions, such as, what is the (global) effect size of PA on cognition, depression, or mobility in advanced age?
To develop specific justifiable exclusion criteria at the study level, and to address all groups of older adults at the research program level. For example, the PREDICT Charter [39] for exploring barriers to including older adults in clinical trials, and even more importantly, proposing specific guidelines and work packages for increasing their participation in research. A more recent example is a document published by the American Food and Drugs Administration (FDA) [72], entitled “Enhancing the Diversity of Clinical Trial Populations – Eligibility Criteria, Enrollment Practices, and Trial Designs; Draft Guidance for Industry; Availability.” In this document, the FDA encourages sponsors of clinical trials to incorporate broader eligibility criteria, when scientifically and clinically appropriate, to increase the enrollment of underrepresented populations in their clinical trials” [72]. This approach should be adopted by research bodies such as journals that focus on PA in old age. Enhancing the diversity of clinical trial populations will enlarge existing databases on older adults and create more solid ground for applying the EBM approach for training older adults. Importantly, PA for functionally impaired older adults may involve safety issues, and consequently ethical barriers. Given the benefits of PA for all levels of functioning, it is the role of a diverse group of scientists to offer guidelines for PA for all older adults, regardless of their levels of functioning.
To replicate current laboratory-based studies regarding the effect of PA on physical and psychological health in real-world settings, while applying rigorous and clinically relevant naturalistic research. For example: two fairly recent studies described an effective personalized exercise program conducted via mobile phones for 52 healthy older adults [52] and via tablets for 40 pre-frail older adults [53]. The success of these experiments should be examined in large groups of older adults in different settings. One way to do so could be to collaborate with stakeholders such as health insurance companies (e.g., [73]), or nursing homes (e.g., [74]).
(a) To increase the utilization of technology-assisted PA interventions, especially for promoting PA in older adults, while measuring the effect of PA on a range of aspects using widely accepted validation protocols. The first recommended steps would be to review and map all available assisting technologies related to PA performance and promotion, examine psychological factors related to the use of each technology in advanced age, and create toolkits for stakeholders and health practitioners that include guidelines on how to optimally utilize the technology for this specific age group.
  (b) To advance the development of self-adapted AI models, including multi-omics profiling combined with clinical measures, as a means for evaluating health status, level of fitness, and other important markers.
  (c) To share data by establishing open data sources related to PA and health in old age.

### The recently established network on evidence-based PA in old age

To implement these suggestions, the European Group for Research into Elderly and Physical Activity (EGREPA) recently initiated a network, funded by the European Cooperation in Science and Technology (COST). Indeed, the “Network on evidence-based physical activity in old age” (PhysAgeNet; CA 20,104) is open for scientists and practitioners who wish to contribute to achieving these aspiring goals. It is up to all of us to accept this challenge and work in collaboration to provide applicable results.

## Data Availability

Not applicable.
